# Multi-omics profiling reveals *Poria cocos* polysaccharides mitigate PEDV-induced intestinal injury by modulating lipid metabolism in piglets

**DOI:** 10.1186/s40104-025-01211-y

**Published:** 2025-05-20

**Authors:** Qian Zhang, Shuaijie Wang, Mengjun Wu, Zihan Tan, Tao Wu, Dan Yi, Lei Wang, Di Zhao, Yongqing Hou

**Affiliations:** Hubei Key Laboratory of Animal Nutrition and Feed Science, Wuhan Polytechnic University, Wuhan, 430023 China

**Keywords:** Intestinal injury, Lipid metabolism, Piglets, Porcine epidemic diarrhea virus, *Poria cocos* polysaccharides

## Abstract

**Background:**

Porcine epidemic diarrhea virus (PEDV) infection poses a significant challenge to the swine industry, with limited effective control measures available. *Poria cocos* polysaccharides (PCP) is the primary active ingredient of *Poria cocos,* and has been demonstrated to show beneficial effects on intestinal damage in previous studies. However, its mechanism has not been fully understood. In the present study, 18 seven-day-old piglets were divided into 3 groups: Control group, PEDV group, and PCP + PEDV group. After three days of adaptation, piglets in the PCP + PEDV group were orally administered 10 mg/kg body weight/d PCP from d 4 to 10. On d 8, piglets were orally administered with PEDV at the dose of 10^4.5^ TCID_50_/piglet. This study aimed to investigate the potential effects of PCP on PEDV-induced intestinal injury and explored the underlying mechanisms.

**Results:**

The results showed that PCP administration effectively alleviated diarrhea, reduced PEDV replication in the small intestine and colon of piglets, and significantly improved intestinal mucosal morphology. Specifically, PCP increased the villus height in both the jejunum and ileum and increased the villus height to crypt depth ratio in the ileum (*P* < 0.05). Improved intestinal function was further evidenced by elevated plasma D-xylose levels and decreased diamine oxidase activity (*P* < 0.05). Transcriptomic and proteomic analyses revealed that lipid metabolism is a key pathway regulated by PCP during PEDV infection. Notably, PCP significantly upregulated sphingolipid metabolism-related genes, including ectonucleotide pyrophosphatase/phosphodiesterase family member 7 and *N*-acylsphingosine amidohydrolase 2. Metabolomic analysis revealed that PCP primarily modulated the levels of plasmanylphosphoethanolamine, lysophosphatidylcholine, and carnitine. Additionally, PCP reversed the expression of key genes involved in fatty acid uptake, intracellular lipid transport, and fatty acid synthesis, such as fatty acid binding protein 2, fatty acid transport protein 4, apolipoprotein B, apolipoprotein C3, fatty acid synthase, long-chain fatty acyl CoA synthetase 3, lipoprotein lipase and acyl-CoA thioesterases 12 (*P* < 0.05).

**Conclusions:**

These findings demonstrate that PCP mitigates PEDV-induced intestinal injury by modulating lipid metabolism and highlight its potential as a dietary supplement for enhancing anti-PEDV defenses and promoting intestinal health in piglets.

## Background

Porcine epidemic diarrhea virus (PEDV) is a highly contagious viral pathogen responsible for porcine epidemic diarrhea (PED), a severe enteric disease that affects pigs worldwide. The virus primarily infects epithelial cells in the small intestine, leading to severe diarrhea, dehydration, and high mortality rates, particularly in piglets. The global pig industry has experienced substantial economic losses due to PED outbreaks [1]. PEDV replication induces significant cellular stress, triggering inflammatory responses in epithelial cells and disrupting the integrity of the intestinal barrier [2]. Additionally, PEDV activates apoptotic pathways, leading to the loss of functional intestinal epithelial cells and further compromising intestinal integrity and function. The resulting villous atrophy impairs nutrient absorption and exacerbates fluid and electrolyte malabsorption, thereby worsening symptoms such as diarrhea and dehydration [3]. To mitigate the impact of PEDV, pig farms have adopted enhanced biosecurity measures and immunization strategies to bolster the resilience of pig populations against the virus [4]. However, PEDV’s high genetic variability and the limited duration of immune protection following vaccination present ongoing challenges for disease prevention and control.

Plant polysaccharides are large molecular compounds abundantly found in plants and are the principal water-soluble active constituents in traditional Chinese herbal decoctions. *Poria cocos*, commonly known as "Fuling" in China, is an edible medicinal mushroom derived from the dried sclerotium of polypore fungi. This fungus contains various bioactive compounds, including polysaccharides, triterpenoids, sterols, amino acids, choline, histidine, and potassium salts. Notably, *Poria cocos* polysaccharides (PCP), which constitute 70%–90% of the dry weight of *Poria cocos*, are recognized as its primary active ingredient [5]. PCP exhibits diverse biological activities, including immunomodulation, anti-inflammatory, antioxidant, and antiviral properties, suggesting potential therapeutic applications [5]. Previous research has indicated that PCP can modulate gastrointestinal function, strengthen the intestinal physical, immune, and microbial barriers [6], and alleviate cisplatin-induced intestinal damage and inflammation [7]. Moreover, PCP has been shown to facilitate intestinal mucosal repair by modulating the TLR and JNK signaling pathways, providing relief from clinical symptoms associated with functional dyspepsia [8]. Given these findings, we hypothesize that PCP may exert beneficial effects on intestinal damage caused by PEDV infection in piglets. Therefore, this study aimed to investigate this hypothesis and elucidate the underlying mechanisms.

## Materials and methods

### Animal experiments

Eighteen 7-day-old crossbred (Duroc × Landrace × Large White) healthy piglets with similar body weight (2.6 ± 0.3 kg) were used in this study. The piglets were randomly assigned to three groups (*n* = 6 per group; half males and half females): control, PEDV, and PCP + PEDV. The trial lasted for 11 d, with the first 3 d serving as an adaptation period. All the piglets were fed a milk replacer ad libitum (milk replacer-to-water ratio of 1:5, water temperature: 45–55 °C). From d 4 to 10, piglets in the PCP + PEDV group were orally administered PCP (10 mg/kg body weight). Piglets in the control and PEDV groups received an equivalent volume of prepared milk replacer. On the evening of d 8, piglets in the PEDV and PCP + PEDV groups were orally inoculated with PEDV at a dose of 10^4.5^ TCID_50_ as described in our previous study [9]. Each pig received 1 mL of the virus mixture, which was dissolved in 2 mL of PBS solution, for a total volume of 3 mL. The control group received an equal volume of PBS. On the evening of d 10, all piglets were deprived of feed and water. On the morning of d 11, the piglets were weighed after they had fasted. Each piglet was then orally administered D-xylose (0.1 mg/kg body weight), and blood samples were collected from the anterior vena cava one-hour later, Subsequently, the piglets were anesthetized, euthanized, and sampled. Tissues, including the duodenum, jejunum, ileum, and colon, were collected, rapidly frozen in liquid nitrogen, and stored at −80 °C for further analysis.

### Feeding management

The trial was conducted in the Level II Biosafety Laboratory at the Wuhan Institute of Animal Husbandry and Veterinary Medicine. Before the trial, the pig pens, cages, walls, and floors were thoroughly cleaned, disinfected, and fumigated. Throughout the trial, the environmental conditions were maintained at 26–28 °C with adequate ventilation. To prevent cross-contamination, piglets in the control and PEDV groups were housed in separate sheds under identical conditions. The milk replacer was provided five times daily, with thorough disinfection of the equipment and pens at each feeding. The pig sheds were cleaned twice daily using alternating disinfectants (povidone-iodine, glutaraldehyde, and benzalkonium bromide). Piglets were monitored daily for feeding behavior, general health, diarrhea, and illness symptoms, with detailed records maintained. Fecal scores were assessed based on the following criteria: Score 0: normal, well-formed feces. Score 1: mildly soft feces, slightly wet but still formed. Score 2: loose, unformed feces, noticeably wet. Score 3: watery diarrhea, no solid matter present.

### Preparation of intestinal tissue sections

A 3-cm segment was collected from the middle portion of the duodenum, jejunum, ileum, and colon. Then they were immediately placed on ice, and the intestinal contents were gently removed with cold phosphate-buffered saline (PBS, pH 7.4) without damaging the villi. The tissues were then fixed in 4% paraformaldehyde for 24 h, embedded in paraffin, and stained with hematoxylin and eosin. All histological procedures from fixation through staining were conducted by Powerful Biology, Inc. (Wuhan, China). Villous and crypt parameters were measured, and the villous height-to-crypt depth (VH:CD) ratio was calculated for the jejunum. Ten intact villi with optimal morphology were selected per sample for quantitative analysis using an Olympus BX-41 TF microscope (Olympus, Tokyo, Japan) and Image-Pro Plus 6.0 software (Media Cybernetics, Rockville, MD, USA).

### Plasma biochemical parameters

Plasma biochemical parameters, including total cholesterol (TC), triglyceride (TG), low-density lipoprotein (LDL), high-density lipoprotein (HDL), creatinine (CREA), blood urea nitrogen (BUN), creatine kinase (CK) and alkaline phosphatase (ALP), were analyzed using a Hitachi 7100 automatic biochemical analyzer. The measurements were repeated three times on the same day to ensure accuracy.

The plasma D-xylose level and diamine oxidase (DAO) activity were determined using reagent kits purchased from Nanjing Jiancheng Bioengineering Institute (catalog numbers: D-xylose, A035-1-1; DAO, A088-1-1).

### Transcriptome sequencing and analysis

Transcriptomic analysis was performed by Shanghai Majorbio Bio-Pharm Technology Co., Ltd. (Shanghai, China) with six biological replicates per group. Total RNA was extracted from jejunum tissues using TRIzol reagent. mRNA was enriched using Oligo(dT) beads, reverse-transcribed into cDNA, and sequenced on an Illumina HiSeq 2500 platform. Gene expression levels were normalized using the fragments per kilobase of transcript per million mapped reads (FPKM) method. Differentially expressed genes (DEGs) were identified based on a fold change ≥ 2 and adjusted *P*-value < 0.05. Gene Set Enrichment Analysis (GSEA) was conducted using the Gene Denovo Omicshare platform (http://www.omicshare.com/tools). Gene Ontology (GO) and Reactome pathway enrichment analyses were performed using Metascape database, while Kyoto Encyclopedia of Genes and Genomes (KEGG) pathway analysis was carried out using DAVID database.

### Tandem Mass Tag (TMT) proteomic and analysis

The tissue samples were lysed in a lysis buffer composed of 8 mol/L urea, 1% SDS, 0.1% PMSF, and 65 mmol/L DTT. The lysates were sonicated on ice for 30 min and centrifuged at 14,000 r/min for 30 min at 4 °C. The protein concentration was quantified using the bicinchoninic acid assay. A total of 100 μg of protein was suspended in 50 μL of buffer containing 100 mmol/L TEAB and 10 mmol/L TCEP and incubated at 37 °C for 1 h. Alkylation was performed by adding 40 mmol/L iodoacetamide, followed incubation at 37 °C for 40 min in the dark. The samples were then precipitated with 300 μL of prechilled acetone, and the resulting precipitate was subjected to trypsin digestion overnight at 37 °C.

The TMT reagents were subsequently added to the resulting peptides, which were then incubated for 2 h at room temperature. The samples were then subjected to LC-MS/MS analysis using an Orbitrap Exploris 480 mass spectrometer coupled to an EASY-nLC 1200 system (Thermo Fisher Scientific, MA, USA). Peptides were chromatographically separated using an Acclaim PepMap C18 analytical column (75 μm × 25 cm, Thermo Fisher Scientific, MA, USA) before being introduced into the Orbitrap Exploris 480 mass spectrometer for analysis. Data acquisition was performed in the data-dependent acquisition mode, automatically switching between MS and MS/MS modes.

The raw data from the TMT-based proteomic analysis were processed and analyzed using Spectronaut X software from Biognosys AG, Switzerland. The software was configured to search the *Sus scrofa* database along with contaminant databases. Differentially expressed proteins (DEPs) were filtered based on a criterion of |fold change| ≥ 1.2 and an adjusted *P*-value < 0.05. The identified DEPs were further analyzed using Reactome gene set enrichment through the online Metascape database. Correlation analysis of the transcriptomic and proteomic data was performed via the Majorbio platform (https://cloud.majorbio.com/page/tools/).

### Plasma metabolomic analysis

Fifty microliters (50 μL) of plasma were mixed with 300 μL of pure methanol, vortexed for 10 s, and then centrifuged at 12,000 r/min for 10 min at 4 °C. The supernatant was collected and centrifuged again at 12,000 r/min for an additional 5 min at 4 °C. Following refrigeration at −20 °C for 30 min, the sample was centrifuged again at 12,000 r/min at 4 °C for 3 min, and 150 μL of the supernatant was transferred to an injection vial for analysis. The sample extracts were then analyzed using a liquid chromatography-electrospray ionization-tandem mass spectrometry (LC-ESI-MS/MS) system, equipped with a Waters ACQUITY UPLC HSS T3 C18 column, under specific analytical conditions. The mass spectrometry analysis was performed in both positive and negative ion modes, with multiple reaction monitoring transitions monitored for each period based on the metabolites eluted within that period.

### Real-time PCR

PEDV *N* gene expression was quantified in intestinal tissues using ribosomal protein L4 (*RPL4*) as a reference gene. RNA was extracted using RNAiso Plus, reverse-transcribed with the PrimeScript RT Reagent Kit with gDNA Eraser according to the manufacturer’s protocol. After cDNA synthesis, real-time PCR was performed using the SYBR Premix Ex Taq™ (Tli RNaseH Plus) reagent. All the reagent kits were purchased from Takara Biotechnology Co., Ltd. (Dalian, China). Relative gene expression levels were calculated and statistically analyzed using the 2^−^^ΔΔCt^ method. The primers used in the present study are listed in Table 1.

**Table 1:** Primer sequences used in the present study

Genes	Forward (5'-3')	Reverse (5'-3')
PEDV *N*	CGCAAAGACTGAACCCACTAACTT	TTGCCTCTGTTGTTACTCGGGGAT
*RPL4*	GAGAAACCGTCGCCGAAT	GCCCACCAGGAGCAAGTT
*FABP2*	AGATAGACCGCAATGAGA	TCCTTCTTGTGTAATTATCATCAGT
*FATP4*	CATGGAAAACTGTAATGAGTTCGTG	CCGCAGGTTGGTGTTGATG
*APOA1*	CCTTGGCTGTGCTCTTCCTC	ACGGTGGCAAAATCCTTCAC
*APOB*	TGGTGGTGGAGACACACACA	CCAACCAGAGACCCATCCA
*APOC3*	CTAACCAGCGTGAAGGAGTC	CAGAAGTCGGTGAACTTGCC
*FASN*	ACACCTTCGTGCTGGCCTAC	ATGTCGGTGAACTGCTGCAC
*ACSL3*	TTTTGCTGTCCCGTTGGTC	GTATCCACCTTCTTCCCAGTTCTTT
*LPL*	AGCCTGAGTTGGACCCATGT	CTCTGTTTTCCCTTCCTCTCTCC
*ACOT12*	CACGAAGAAGGCCTCTCAAA	GGATGAGAAATCCGGCACATA

### Statistical analysis

Experimental data, including average body weight, fecal scores, and plasma biochemistry, were analyzed using SPSS 20.0 software. One-way ANOVA followed by Duncan’s multiple comparison test was applied. The results are presented as mean ± standard deviation (SD), and *P* < 0.05 was considered statistically significant. Bars not sharing a common lowercase letter in figures differ significantly (*P* < 0.05).

## Results

### Effects of PCP on the growth performance and viral gene expression in piglets infected with PEDV

**Fig. 1:**
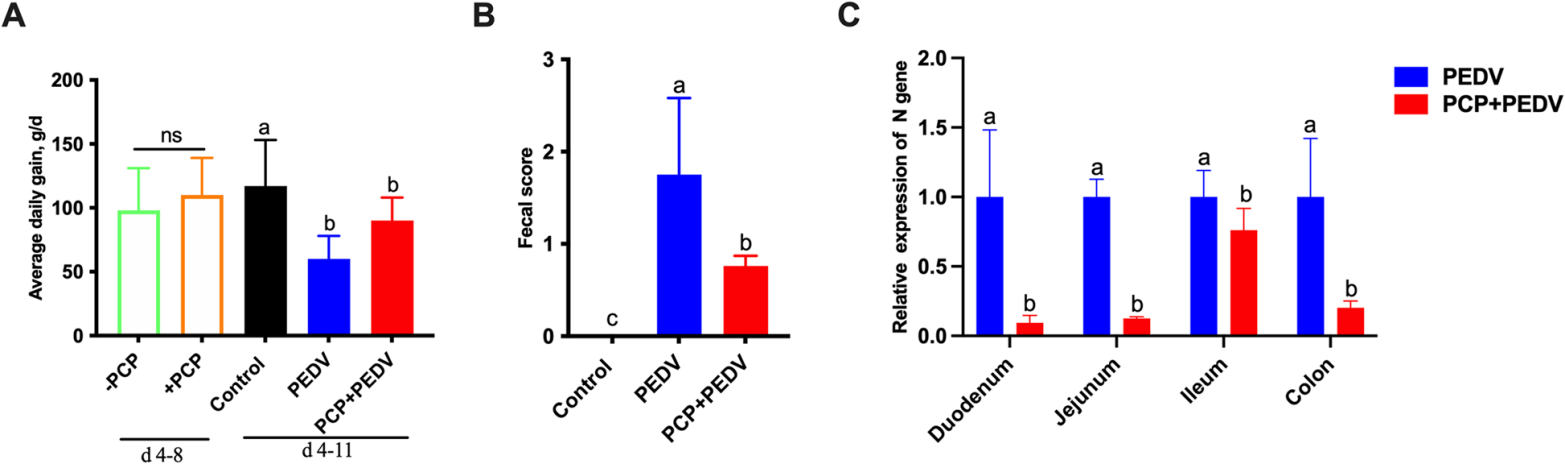
Effects of PCP on the growth performance (**A**), fecal score (**B**), and viral gene expression (**C**) in PEDV-infected piglets. From d 4 to 10, piglets in the PCP + PEDV group received oral PCP (100 mg/kg BW/d). On d 8, piglets in the PEDV and PCP + PEDV groups were orally administered PEDV at a dose of 1 × 10^4.5^ TCID_50_ per piglet. Body weights were recorded, and diarrhea incidence was monitored three times daily. The relative expression of the PEDV *N* gene in the small intestine was quantified using real-time PCR. The data are presented as mean ± SEM (*n* = 6). Bars not sharing a common lowercase letter differ significantly (*P* < 0.05)

Prior to viral infection, there were no significant differences in average daily gain (ADG) between the PCP-administered and control groups. However, after viral infection, the ADG of PEDV group was significantly lower than that of the control group (*P* < 0.05). Conversely, the PCP + PEDV group showed a slight, albeit statistically insignificant, improvement in ADG compared to the PEDV group (*P* > 0.05) (Fig. 1A). Moreover, PEDV infection significantly increased the fecal score, while PCP administration effectively mitigated diarrhea severity (*P* < 0.05) (Fig. 1B).

Following viral infection, PEDV was detected in both the small intestine and colon of piglets. Notably, PCP administration significantly reduced viral gene expression levels in intestinal regions when compared to the PEDV-infected group (*P* < 0.05) (Fig. 1C).

### Effects of PCP on plasma biochemical parameters in PEDV-infected piglets

**Fig. 2:**
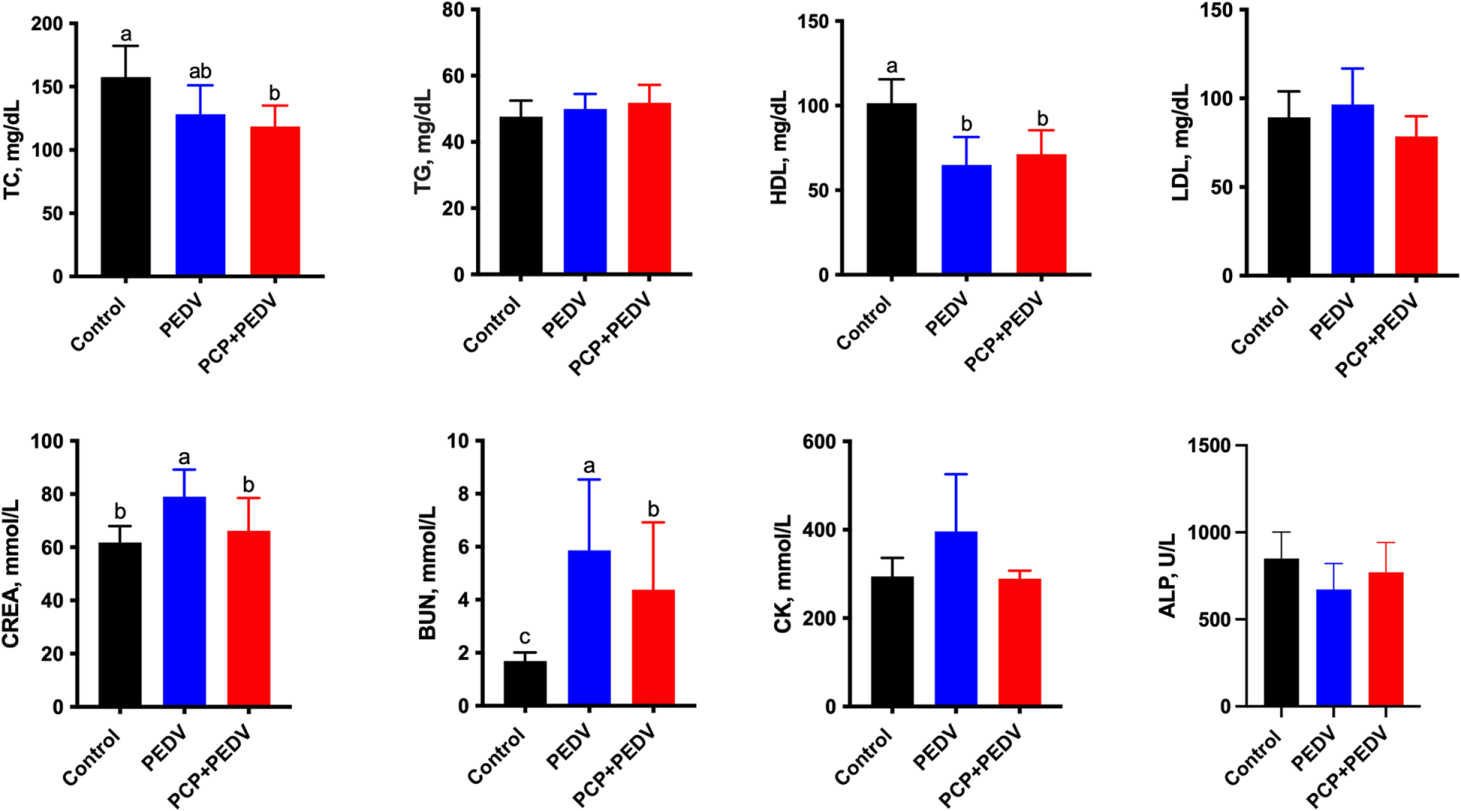
Effects of PCP on plasma biochemical parameters in PEDV-infected piglets. Plasma biochemical parameters were measured on the day of blood collection and the measurements were repeated three times for accuracy. TC: total cholesterol; TG: triglyceride; HDL: high-density lipoprotein; LDL: low-density lipoprotein; CREA: creatinine; BUN: blood urea nitrogen; CK: creatine kinase; ALP: alkaline phosphatase. The data are presented as mean ± SEM (*n* = 6). Bars not sharing a common lowercase letter differ significantly (*P* < 0.05)

Compared to the control group, PEDV infection significantly reduced plasma HDL levels and increased CREA and BUN levels (*P* < 0.05) (Fig. 2). A decreasing trend was also observed in total TC levels (*P* > 0.05). In comparison to the PEDV-infected group, PCP administration significantly reduced CREA and BUN levels in the PEDV-infected group (*P* < 0.05). Additionally, PCP partially reversed PEDV-induced changes in CK and ALP levels, although these effects were not statistically significant (*P* > 0.05).

### Effects of PCP on the intestinal function and morphological structure of the small intestine in PEDV-infected piglets

**Fig. 3:**
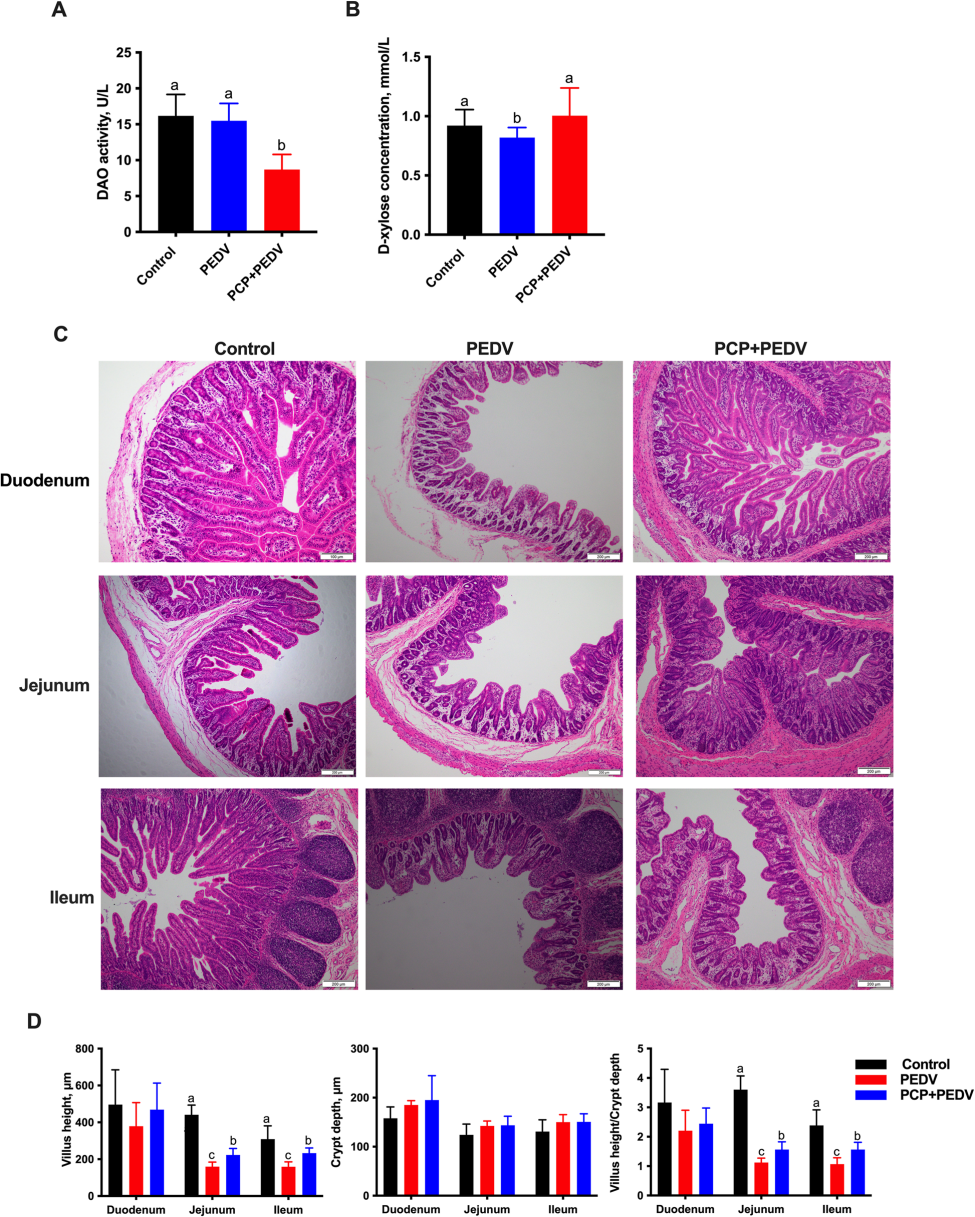
Effects of PCP on intestinal function and small intestine morphology in PEDV-infected piglets. **A** and **B** Plasma D-xylose levels (**A**) and diamine oxidase (DAO) activity (**B**) were determined using commercial kits. **C** Representative images of intestinal morphology after staining with hematoxylin and eosin (× 100 magnification). **D **Villus height (tip to crypt mouth) and crypt depth (crypt mouth to base) were measured, and villus height/crypt depth was calculated. The data are presented as mean ± SEM (*n* = 6). Bars not sharing a common lowercase letter differ significantly (*P* < 0.05)

In comparison to the control group, the PEDV group exhibited a significant decrease in plasma D-xylose levels (*P* < 0.05) (Fig. 3A). However, compared to the PEDV group, the administration of PCP significantly increased plasma D-xylose levels (*P* < 0.05) while significantly reduced DAO levels (*P* < 0.05) (Fig. 3A and B).

Morphological analysis revealed villus atrophy and shortening in the small intestines of PEDV-infected piglets, which were significantly ameliorated by PCP administration (Fig. 3C). Villus height and the villus height-to-crypt depth (VH:CD) ratio in both the jejunum and ileum were significantly lower in the PEDV group (*P* < 0.05) than in the control group (*P* < 0.05) (Fig. 3D). However, compared to the PEDV-infected group, PCP administration significantly improved both the villus height and the VH:CD ratio in these regions (*P* < 0.05).

### Transcriptome analysis

**Fig. 4:**
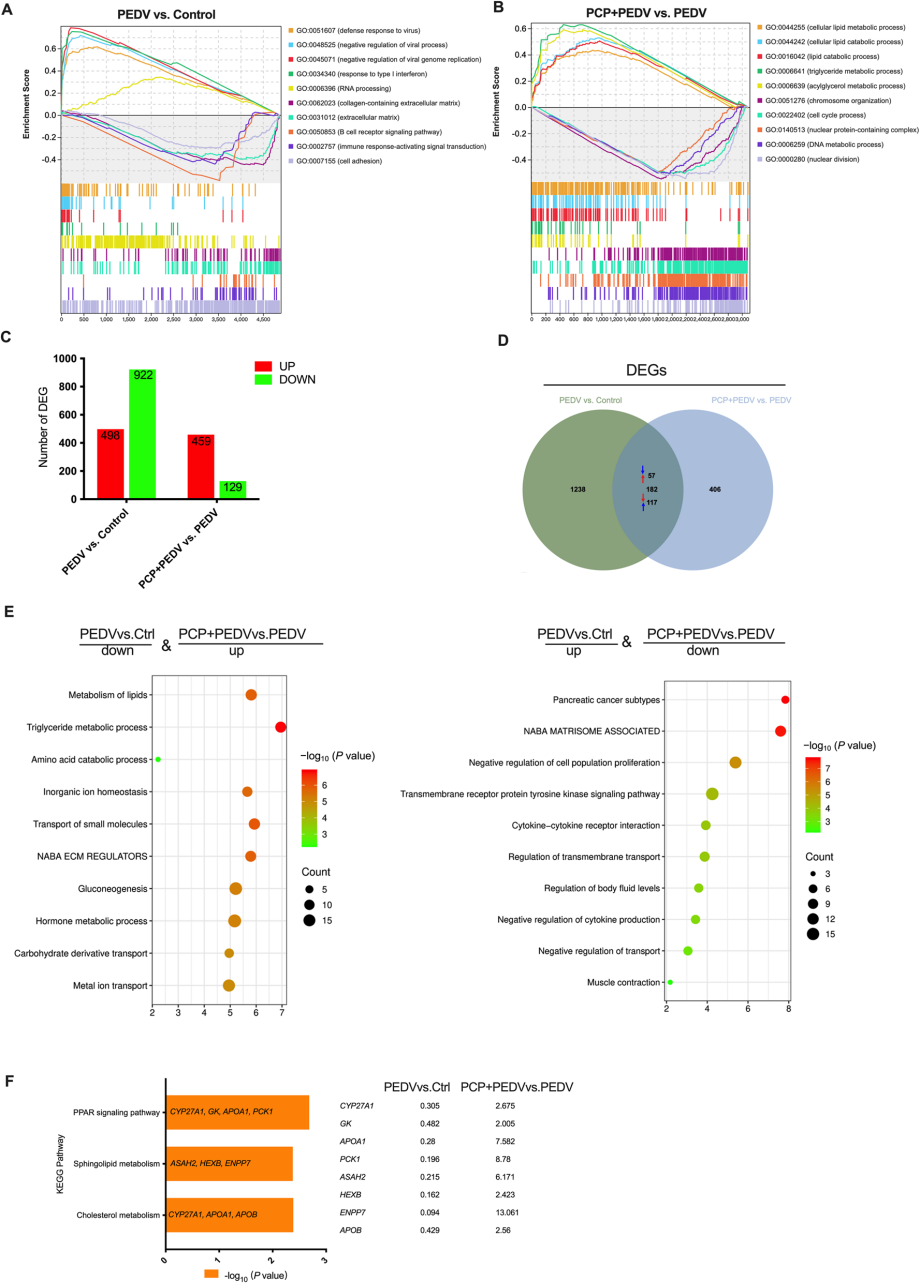
Transcriptomic analysis of PEDV-infected piglets treated with PCP. **A** and **B** The top five positively and negatively enriched gene sets for PEDV infection (PEDV vs. control) and PCP treatment (PCP + PEDV vs. PEDV) identified by GSEA, sorted by normalized enrichment score (NES). **C** Overview of differentially expressed genes (DEGs; fold change > 1.5, *P* < 0.05) in the jejunum. **D** Venn diagram showing overlapping DEGs between groups, with arrows indicating genes showing opposite trends. **E** Biological process enrichment analysis of reversed genes using Metascape. **F** Lipid metabolism pathways and genes modulated by PCP treatment were analyzed using the DAVID database. The fold changes of genes are indicated

GSEA was initially performed on all genes that met the criterion of an adjusted *P*-value ≤ 0.05, irrespective of the fold change in the comparative groups. Following PEDV infection, the top five significantly enriched and upregulated biological processes were involved primarily in viral defense mechanisms and the type I interferon response, whereas the top five downregulated biological processes were associated with the extracellular matrix, B cell receptor signaling pathway, and immune response-activating signal transduction (Fig. 4A). Compared with those in the PEDV group, the lipid metabolism-related gene sets in the PCP + PEDV group were significantly enriched and upregulated (Fig. 4B).

Using the criteria of |fold change| ≥ 2 and adjusted *P*-value ≤ 0.05, 498 upregulated and 922 downregulated genes were identified in the PEDV vs. control comparison (Fig. 4C). In contrast, 459 genes were upregulated and 129 were downregulated in the PCP + PEDV vs. PEDV comparison. A total of 182 DEGs overlapped between the two comparisons (Fig. 4D). Among these genes, 57 genes upregulated in the PEDV group vs. Control group were downregulated in the PCP + PEDV group vs. PEDV group, while 117 genes downregulated in the PEDV group were upregulated following PCP administration.

Enrichment analysis of the 117 upregulated genes revealed their involvement in pathways related to metabolism and substance transport, including “metabolism of lipids”, “triglyceride metabolic process”, “amino acid catabolic process”, “inorganic ion homeostasis,” and “transport of small molecules” (Fig. 4E). Conversely, the 57 downregulated genes were primarily associated with immune response pathways, such as the “transmembrane receptor protein tyrosine kinase signaling pathway”, “cytokine-cytokine receptor interaction”, and “negative regulation of cytokine production” (Fig. 4E). KEGG pathway analysis further revealed that the PCP-upregulated genes were enriched in lipid metabolism pathways, including the PPAR signaling pathway, sphingolipid metabolism, and cholesterol metabolism (Fig. 4F). Notably, significant changes were observed in sphingolipid metabolism-related genes, such as *N*-acylsphingosine amidohydrolase 2 (*ASAH2*), hexosaminidase subunit beta (*HEXB*), and ectonucleotide pyrophosphatase/phosphodiesterase family member 7 (*ENPP7*).

### Proteomic analysis

**Fig. 5:**
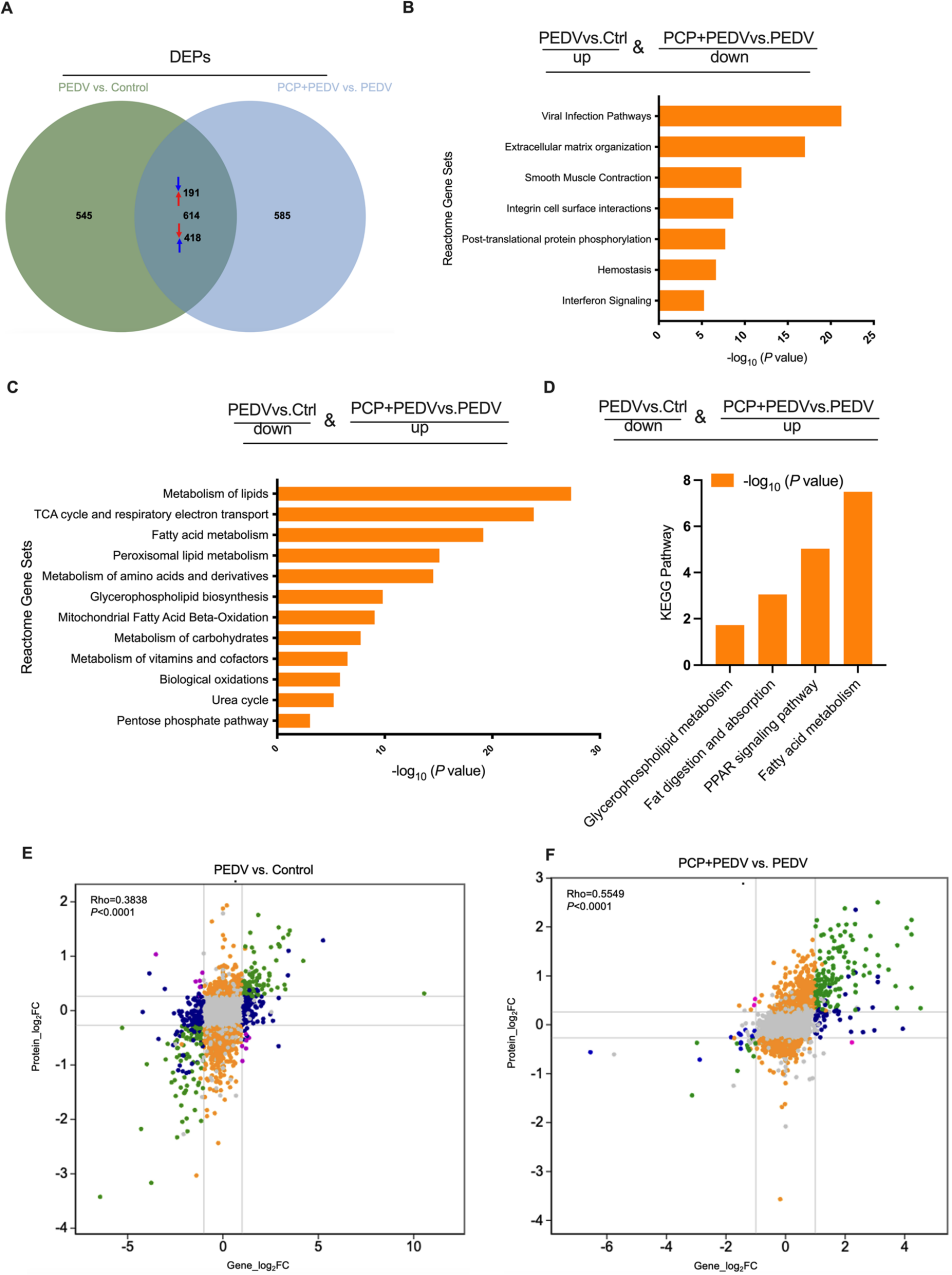
Proteomic analysis of PEDV-infected piglets treated with PCP. **A** Venn diagram illustrating overlapping proteins between groups, with arrows indicating proteins showing opposite trends. **B** and **C** Enrichment analysis of reversed proteins using Metascape. **D** KEGG pathway analysis of upregulated proteins using DAVID. **E** and **F** Correlation analysis between the transcriptomic and proteomic data

Using the criteria of |fold change| ≥ 1.2 and adjusted *P*-value ≤ 0.05, 1,160 DEPs and 1,199 DEPs were identified in the PEDV vs. control and PCP + PEDV vs. PEDV comparisons, respectively. Among these, 614 proteins were common (Fig. 5A), most of which were also reversed by PCP treatment. Specifically, 191 proteins upregulated in the PEDV group vs. control group were downregulated in the PCP + PEDV group vs. PEDV group, while 418 downregulated proteins in the PEDV group were upregulated by PCP. Reactome pathway analysis revealed enrichment of viral infection pathways for downregulated proteins (Fig. 5B) and lipid metabolism pathways for upregulated proteins (Fig. 5C). KEGG analysis further supported enrichment in lipid metabolism pathways (Fig. 5D). The Pearson correlation coefficients of 0.3838 and 0.5549 indicated strong agreement between the transcriptomic and proteomic datasets (Fig. 5E and F).

### Metabolomic analysis

**Fig. 6:**
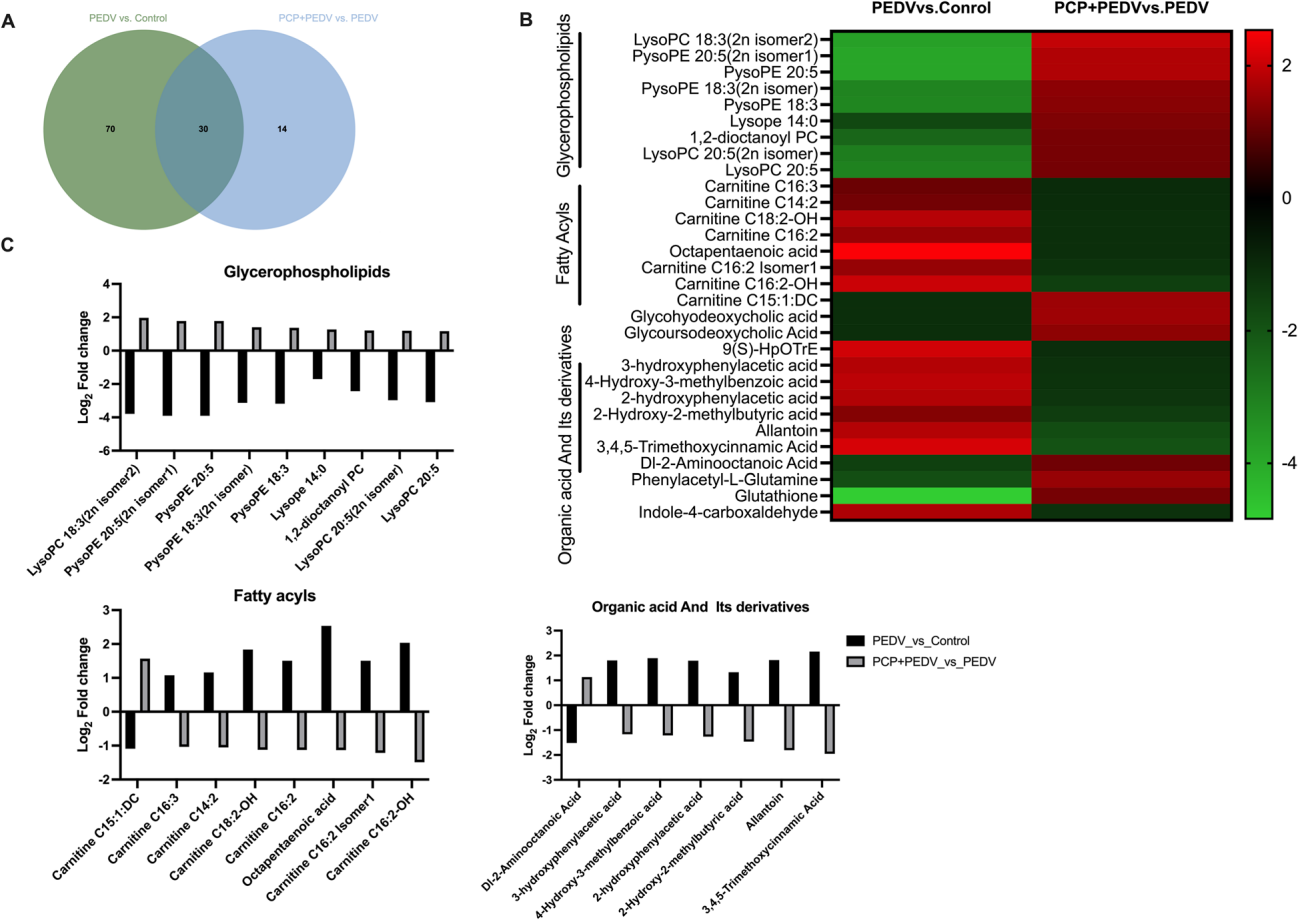
Metabolomic analysis of PEDV-infected piglets treated with PCP. **A** Venn diagram illustrating overlapping metabolites between groups. **B** Heatmap showing reversed metabolites across the comparison groups. **C** Bar graph showing the fold changes of key metabolites between the groups

Transcriptomic and proteomic data suggest that PCP mitigates PEDV-induced lipid metabolism disturbances. To identify specific metabolites involved, metabolomic analysis was performed. Using the criteria of |fold change| ≥ 2 and VIP ≥ 1, 100 and 44 metabolites were identified in the PEDV vs. control and PCP + PEDV vs. PEDV comparisons, respectively. Among them, 30 metabolites were common between the comparisons and presented opposite expression patterns (Fig. 6A). These metabolites can be classified into three main categories: glycerophospholipids, fatty acyls, and the organic acid and its derivatives (Fig. 6B). PEDV infection significantly reduced plasmanylphosphoethanolamine (PysoPE) and lysophosphatidylcholine (LysoPC) levels in the glycerophospholipid category, which were restored by PCP treatment (Fig. 6C). Conversely, most carnitines in the fatty acyls category exhibited opposite patterns.

### Effects of PCP on lipid metabolism-associated genes in the jejunum

**Fig. 7:**
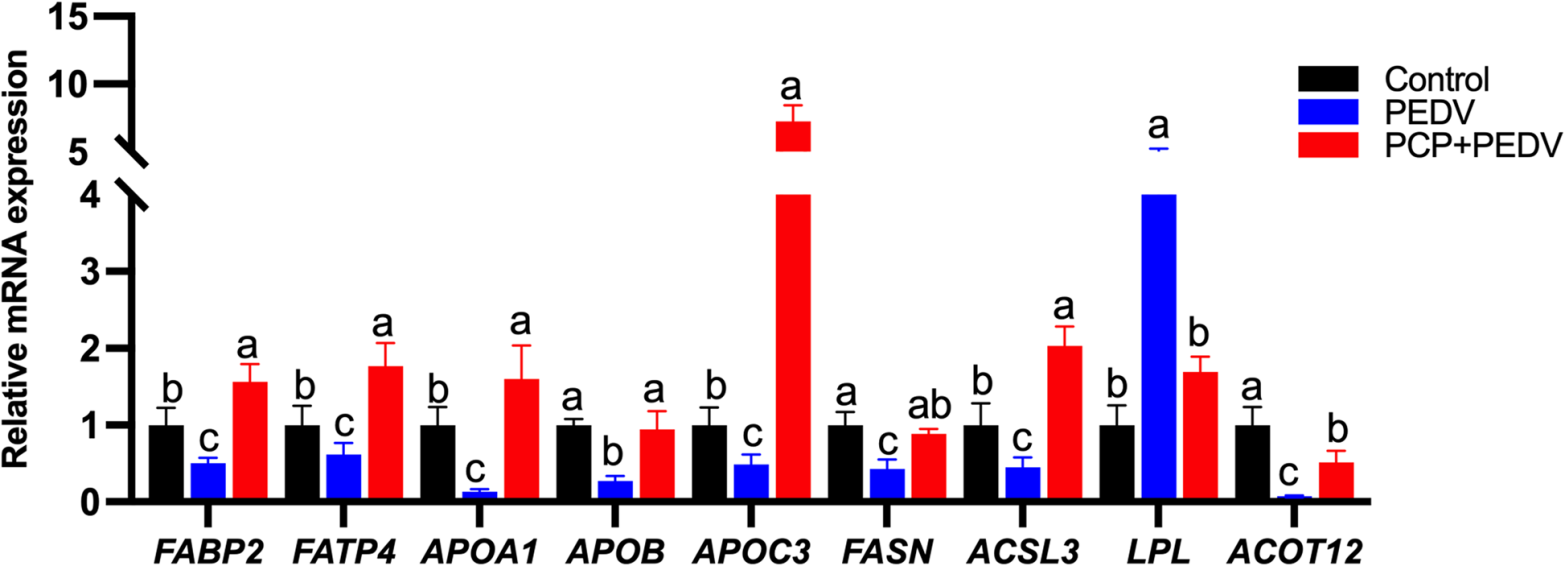
Effects of PCP on lipid metabolism-associated gene expression in the jejunum of PEDV-infected piglets. The expression levels of lipid metabolism-associated genes were quantified using real-time PCR. The data are presented as mean ± SEM (*n* = 6). Bars not sharing a common lowercase letter within each gene differ significantly (*P* < 0.05)

PEDV infection significantly downregulated lipid metabolism-related genes, including fatty acid binding protein 2 (*FABP2*), fatty acid transport protein 4 (*FATP4*), apolipoprotein A1 (*APOA1*), apolipoprotein B (*APOB*), apolipoprotein C3 (*APOC3*), fatty acid synthase (*FASN*), long-chain fatty acyl CoA synthetase 3 (*ACSL3*) and acyl-CoA thioesterases 12 (*ACOT12*). In contrast, the expression of lipoprotein lipase (*LPL*) was significantly increased. Notably, PCP administration restored the expression of all the above genes (Fig. 7).

## Discussion

It is well established that viruses intricately manipulate host metabolic networks to support their own survival. These interactions disrupt metabolic pathways, leading to disturbances in the host's physiological homeostasis [10]. Therefore, effective interventions must target these dysregulated metabolic pathways that contribute to disease progression. In this study, we demonstrated that PCP significantly inhibits PEDV replication in the intestines, promotes intestinal healing, and alleviates diarrhea. Through multi-omics analyses, we revealed that PCP strongly regulates lipid metabolism, significantly impacting the levels of PysoPE, LysoPC, and carnitine. Further experiments revealed that PCP could promote fatty acid uptake, intracellular lipid transport, and fatty acid synthesis in PEDV-infected piglets. These findings highlight the potential of PCP as a dietary supplement to modulate lipid metabolism, thereby preventing PEDV infection and improving intestinal health in piglets.

In this study, PCP did not significantly affect the daily weight gain of piglets, which contrasts with previous findings [11]. However, PCP substantially reduced PEDV levels in the intestines and alleviated diarrhea. Interestingly, another study found that PCP alone had no effect on the viral load, but when combined with tylvalosin, it exhibited superior efficacy against PRRSV infection compared to tylvalosin alone [12]. Additionally, PCP was found to increase antigen-specific antibody levels and promote splenocyte proliferation in mice immunized with influenza vaccines, demonstrating its role as an effective adjuvant against viral infections, including influenza and Marburg virus [13, 14]. We postulate that the significant decrease in viral gene expression observed in in this study may be attributed to PCP’s ability to boost both humoral and cellular immunity, as evidenced by the transcriptomic and proteomic results. This immune modulation likely contributes to restoring host immune homeostasis.

Elevated CREA and BUN levels commonly indicate renal dysfunction, but establishing a direct connection between PEDV infection and renal dysfunction is challenging. Previous research detected PEDV in the kidney via RT-PCR but not through immunohistochemistry, and no significant pathological changes were observed [15]. Nevertheless, severe diarrhea induced by PEDV can disrupt electrolyte and fluid balance, leading to dehydration and indirectly elevating CREA and BUN levels. Diarrhea also enhances protein metabolism, which accelerates amino acid breakdown and consequently increases urea production, contributing to elevated BUN levels. In this study, PCP administration normalized CREA and BUN levels, likely due to its role in alleviating diarrhea. One possible mechanism is the action of indigestible polysaccharides, which enhance the water-holding capacity of gastrointestinal contents [6].

The presence of DAO in the bloodstream indicates intestinal barrier damage, whereas D-xylose serves as a marker for intestinal absorption capacity. The elevated D-xylose and reduced DAO levels observed in the PCP + PEDV group suggest that PCP could improve both the integrity and function of the intestinal barrier. PCP has been shown to enhance the biochemical barrier by upregulating MUC2, β-defensin, and secretory IgA expression in intestinal tissues [6]. Additionally, PCP can modulate the gut microbiota, increasing the abundance of short-chain fatty acid-producing bacteria and those that increase tight junction protein production [16–18]. These changes strengthen the biological and physical barriers. PCP may also promote epithelial cell proliferation via the Wnt/β-catenin pathway, improving intestinal morphology [6]. Collectively, these findings demonstrate that PCP enhances intestinal barrier function in PEDV-infected piglets.

In this study, the decreased HDL and declining TC levels following PEDV infection suggest a dysregulation of the lipid balance, potentially affecting the energy supply and immune response. Multi-omics analyses further highlight that PCP primarily regulates PEDV infection by modulating lipid metabolism. Metabolomic analysis revealed that PCP influences metabolites related to glycerophospholipids and carnitine. Phosphatidylcholine and phosphatidylethanolamine, two major phospholipids in cell membranes, play crucial roles in maintaining membrane structure and function. Their properties, such as fluidity and curvature, may create favorable conditions for viral infection and replication [19]. LysoPC and PysoPE, primarily resulting from the hydrolysis of phosphatidylcholine and phosphatidylethanolamine by phospholipase A2 (PLA2) [20], presented significantly decreased plasma levels following PEDV infection in this study, suggesting intestinal structural disruption. This finding aligns with a previous hepatitis B study, which demonstrated a negative correlation between LysoPC levels and tissue damage severity [21]. This decrease may result from impaired intestinal absorption, dysregulated phospholipid metabolism, or reduced synthesis due to the limited fatty acids availability during PEDV infection. Interestingly, studies on other coronaviruses, such as HCoV-229E and MERS-CoV, have shown that these viruses stimulate lysophospholipid production to promote the formation of replicative organelles for viral RNA synthesis [22]. It is important to note that these findings were based on in vitro studies, contrasting with the in vivo conditions of this research. In addition to glycerophospholipids, we found that PCP could also influence sphingomyelin content by increasing *ENPP7* and *ASAH2* expression. Sphingomyelin and cholesterol are critical components of lipid rafts, which provide an environment for the aggregation of viral proteins on the cell membrane and facilitate interactions with other membrane proteins, such as receptors [23]. ENPP7 could convert sphingomyelin into ceramide and phosphocholine [24]. Ceramide is further broken down by ASAH2 into sphingosine and fatty acids [25]. Therefore, the increased expression of *ENPP7* and *ASAH2* by PCP administration may lead to the degradation of sphingomyelin, potentially disrupting lipid raft structure and inhibiting PEDV replication. Additionally, the generated sphingosine and ceramide may hinder viral replication by interfering with viral-receptor interactions or by inducing apoptosis [26, 27]. Overall, the regulation of phospholipid metabolism by PCP during PEDV infection may play a crucial role in maintaining intestinal barrier integrity and enhancing antiviral capacity.

Carnitine, an essential compound for transporting fatty acids into mitochondria for β oxidation, was elevated in the plasma of PEDV-infected pigs in this study. Approximately 75% of carnitine is originates from dietary sources [28]. Previous studies have shown that carnitine can elevate serum AMPK levels [29], while AMPK activation induces autophagy and lipophagy, processes known to support PEDV replication [30, 31]. Therefore, the elevated plasma carnitine levels in PEDV-infected pigs suggest that the virus may manipulate host metabolism to enhance energy production and facilitate viral replication. Various viruses, including ASFV, Zika virus, and Dengue virus, have been demonstrated to increase acylcarnitine levels in the host, which has been shown to enhance viral replication [32, 33]. Moreover, carnitine stimulation can suppress host cellular and humoral immune responses, impairing HBV control during chronic infection [34]. In contrast, carnitine treatment reduced intracellular lipid droplets, which are essential for HCV assembly [35]. These findings underscore the complex role of carnitine in viral infections. Further investigation is warranted to fully elucidate its precise role in PEDV infection and how it is related to the ability of PCP to inhibit PEDV replication.

Dietary lipids are digested into fatty acids in the intestinal lumen and are absorbed by enterocytes via passive diffusion or facilitated by transporters such as CD36 and FATP4. Once inside cells, fatty acids are transported by proteins such as FABP1 and FABP2 [36]. FABP1 predominantly binds to fatty acids from the bloodstream for incorporation into mucosal phospholipids or for oxidation, while FABP2 primarily transports dietary fatty acids for triglyceride synthesis and lipoprotein formation [37]. Various apolipoproteins, such as APOB and APOC3, further regulate lipid transport, metabolism, and distribution [38]. In this study, real-time PCR analysis revealed that PCP could modulate the expression of key genes involved in lipid metabolism, including *FATP4* and *FABP2*. These findings suggests that PCP enhances fatty acid uptake and transport in enterocytes, potentially leading to increased expression of *APOB* and *APOC3*, which are beneficial for lipoprotein assembly and secretion. Furthermore, the upregulation of *FASN* and *ACSL3*, and the downregulation of *LPL* may contribute to fatty acid and lipid synthesis in enterocytes, which would help maintain host energy levels and affect viral replication. Research has demonstrated that treating infected enteroids with palmitic acid significantly reduced PEDV replication [39]. However, the re-esterification of fatty acids into lipids may promote PEDV replication, as reported by Shi et al. [40]. Overall, these findings indicate that PCP can regulate fatty acid metabolism in enterocytes, promoting fatty acid uptake, intracellular transport, and synthesis.

This study revealed that PCP effectively improved lipid metabolism disorders induced by PEDV infection. However, the precise mechanisms remain unclear. PCP is a complex carbohydrate composed of ribose, arabinose, xylose, mannose, glucose, and galactose. Its biological activity depends largely on factors such as molecular weight, monosaccharide composition, glycosidic bonds, and overall conformation. Due to their high molecular weight and limited bioavailability, plant polysaccharides are typically resistant to gastrointestinal digestion and enzymatic degradation [5]. In the intestine, PCP may undergo fermentation by the gut microbiota, producing metabolites that interact with intestinal epithelial cells and influence various cellular processes [41]. Additionally, PCP could alter gut microbiota composition and metabolic activity [42]. For example, water-insoluble polysaccharides were shown to enhance anaerobic bacterial abundance, activating the PPAR-γ signaling pathway to maintain gut hypoxia. This process inhibited PGE2-producing fungi and Proteobacteria, thereby reducing hepatic inflammation and fat accumulation [43]. Based on these findings, we speculate that PCP primarily exerts its effects indirectly through modulation of gut microbiota and associated cellular signaling pathways. Further investigations are needed to validate this hypothesis and clarify the role of microbiota in PCP’s regulation of lipid metabolism.

## Conclusions

In conclusion, this study demonstrated that PCP mitigated PEDV-induced intestinal damage and dysfunction. PCP treatment reduced viral replication in the small intestine, alleviated diarrhea, and restored the intestinal structure in infected piglets. Multi-omics analyses revealed that PCP primarily regulated lipid metabolism, significantly influencing the expression of sphingolipid metabolism-related genes (*ENPP7* and *ASAH2*) in the jejunum and the levels of PysoPE, LysoPC, and carnitine in the plasma. PCP also restored fatty acid metabolism disrupted by PEDV infection. Collectively, these findings indicate that PCP can protect against PEDV infection and alleviate associated intestinal injury, with its regulation of lipid homeostasis being a key underlying mechanism.

## Data Availability

All data generated or analyzed during this study are included in this published article. The data during the current study are available from the corresponding author upon request.

## Abbreviations

ADG: Average daily gain
ALP: Alkaline phosphatase
APOA1: Apolipoprotein A1
APOB: Apolipoprotein B
APOC3: Apolipoprotein C3
ACOT12: Acyl-CoA thioesterases 12
ACSL3: Long-chain fatty acyl CoA synthetase 3
ASAH2: *N*-Acylsphingosine amidohydrolase 2
BUN: Blood urea nitrogen
CK: Creatine kinase
CREA: Creatinine
DEGs: Differentially expressed genes
DEPs: Differentially expressed proteins
DAO: Diamine oxidase
ENPP7: Ectonucleotide pyrophosphatase/phosphodiesterase family member 7
FABP2: Fatty acid binding protein 2
FATP4: Fatty acid transport protein 4
FASN: Fatty acid synthase
GO: Gene Ontology
GSEA: Gene Set Enrichment Analysis
HDL: High-density lipoprotein
HEXB: Hexosaminidase subunit beta
KEGG: Kyoto Encyclopedia of Genes and Genomes
LDL: Low-density lipoprotein
LPL: Lipoprotein lipase
PCP: Poria cocos polysaccharides
PEDV: Porcine epidemic diarrhea virus
PysoPE: Plasmanylphosphoethanolamine
RPL4: Ribosomal protein L4
TC: Cholesterol
TMT: Tandem Mass Tag
TG: Triglyceride
VH:CD: Villous height-to-crypt depth
LysoPC: Lysophosphatidylcholine
